# The efficacy of picture vs. word danger cues in reducing imitation of dangerous actions in children

**DOI:** 10.3389/fpsyg.2024.1402021

**Published:** 2024-09-17

**Authors:** Shuanglong Dong, Liang Zhao, Zhaobin Bian, Yansong Wang

**Affiliations:** ^1^School of Teacher Development, Shaanxi Normal University, Xi’an, China; ^2^School of Education, Baoji University of Arts and Sciences, Baoji, China; ^3^School of Music Education, Xi’an Conservatory of Music, Xi’an, China

**Keywords:** imitation effect, dangerous action, word cue, picture cue, children

## Abstract

**Introduction:**

Children are naturally curious and often have limited self-control, leading them to imitate both safe and dangerous actions. This study aimed to investigate whether dangerous cues could effectively inhibit children’s imitation of hazardous behaviors and to compare the effectiveness of picture cues versus word cues in reducing this imitation.

**Methods:**

Seventy-six children were divided into two groups: one group received picture cues, and the other received word cues. Both groups observed an agent grasping an object and were instructed to perform a corresponding keystroke response when a number appeared. A comparable group of adults was also included for reference.

**Results:**

The results demonstrated that picture cues were significantly more effective than word cues in reducing the children’s tendency to imitate dangerous actions.

**Discussion:**

These findings suggest that picture cues are a more effective method for preventing imitation of risky behaviors in children, which has important implications for improving safety education and accident prevention strategies through the use of visual danger cues.

## Introduction

Imitation plays a crucial role in human learning. Through imitation, individuals can modify their personal schema structures to better adapt to new environments. Research indicates that observing an agent performing an action can automatically trigger the corresponding action system in the observer. For instance, when an agent engages in a power grasp., the observer’s power grasp posture is activated, facilitating an imitative action response (Heyes, 2011; Vainio et al., 2007). Behavioral studies related to the action imitation effect have shown a congruency effect between task-unrelated behavioral characteristics and individuals’ behavioral responses during task judgment. These studies employed a stimulus–response congruent task, presenting participants with images of an agent’s actions that were irrelevant to the task. Individual responses were facilitated when the agent’s actions were congruent with the participants’ responses (Brass et al., 2000; Stürmer et al., 2000; Zhao et al., 2023).

However, in daily life, humans do not copy other people indiscriminately. Actually, in some scenarios (e.g., the demonstrator has self-harm behavior), imitation can be inappropriate, and it is essential to circumvent the tendency to imitate (Cross and Iacoboni, 2014). That is, we need to inhibit or control imitation in such specific situations (Bien et al., 2009; Nishimura et al., 2018; van Leeuwen et al., 2009). Darda and Ramsey (2019) conducted a meta-analysis indicated that the control of automatic imitation is guided by brain regions in the multiple demand network including dorsolateral frontoparietal cortex This result supported the role of domain-general control mechanisms in regulating the tendency to imitate.

Recently, Zhao and his colleagues conducted a series of behavioral studies. In these studies, participants were asked to observe a human agent’s left or right hand reaching and grasping a neutral or dangerous object, almost at the same time that the agent finally grabbed the object, the number “1” or “2” appeared on the screen. If the number was ‘1’, participants should press the ‘A’ key on the keyboard with their left index finger; if it was ‘2’, they should press the ‘L’ key on the keyboard with their right index finger. When the object is neutral, the imitation effect appears as the agent’s action hand and the participant’s response hand were on a specular side; while, when the object is dangerous, they restrained the tendency to imitate such dangerous action (Zhao and Sang, 2020; Zhao et al., 2023). Undoubtedly, the suppression of the tendency to imitate dangerous actions has certain survival significance.

Despite the results of Zhao and Sang (2020) that school-age children can adjust their imitation actions according to the perceived danger of actions, compared with adults, it May be more difficult for children to suppress imitation effect, due to their strong curiosity and inadequate control ability (Zhao, 2020). In addition, some researchers believe that children have a strong tendency to imitate human behavior (Stengelin et al., 2023; Whiten et al., 2009), so children are likely to imitate dangerous behavior of others. In order to reduce the tendency of children to imitate dangerous actions, we intend to introduce a warning cue before the agent performs a dangerous action. Myachykov et al. (2013) elaborated that prompt could affect the potential interaction behavior between subjects and objects in a top-down manner. In particular, for school-age children, danger cues enhance their proper operation of dangerous objects, that is, the tendency to avoid dangerous actions (Zhao, 2020).

However, in Zhao (2020), the warning cue is only in the form of words, and the studies on warning showed that pictures have greater advantages compared with words: pictures have the advantage of capturing attention and conveying information quickly (Laughery and Wogalter, 2006), For people who do not have the ability to read or are poor readers, pictures can promote comprehension and better evoke emotional responses (Popova et al., 2018); word processing does not (Glaser and Glaser, 1989), textual stimuli require additional processing before access to their emotional aspects, this processing involves top-down processing, which generates the psychological representation to help us access emotional aspects of stimuli through mental imagery, propositions, or both (Kanske and Kotz, 2007).

In this study, our goal is to investigate how picture and word cues help reduce children’s tendency to imitate dangerous actions. Additionally, like Zhao and Sang (2020), to facilitate the observation of research effects, we used adults as the reference subjects. A digit judgment task was used, and participants were asked to judge the digits that appeared on the screen with the left and right hands (like Zhao and Sang, 2020; Zhao et al., 2023). In our experiments, before the hand of agent gradually approached and grasped a neutral or dangerous object, a safe or dangerous word (in experiment 1) or picture (in experiment 2) cue was present in the first place. We hypothesize that under the influence of the cues, both adults and children were able to suppress the tendency to imitate dangerous actions, and the picture cue was more effective than the text cue.

## Experiment 1

### Method

#### Participants

Seventy six participants were recruited in this experiment. Thirty-eight elementary school students (19 males), aged between 7 and 13 years old, participated in Experiment 1a; 38 college students (20 males), aged between 20 and 25 years old, participated in Experiment 1b. All of the participants were recruited at random. All participants were right-handed, and they had normal or corrected-to-normal eyesight and had not participated in similar experiments.

#### Materials

Stimuli consisted of a series of pictures that depicted an agent’s hand reaching toward and grasping either a neutral or a dangerous object. According to previous studies (Anelli et al., 2012; Hudson et al., 2016), the neutral objects are the cup, spoon, apple, and light bulb; the dangerous objects are the broken cup, dagger, cactus, and broken light bulb. Each picture subtended 0.29° horizontally and 0.72° vertically.

Like Zhao and Sang (2020), to test the validity of this dichotomy, the degree of danger for each set of pictures was evaluated by an independent group of 20 participants (11 females, 9 males; age: 19–24 years). Each set of pictures was rated on a 7-point Likert scale from 1 (not at all dangerous) to 7 (extremely dangerous). Results showed that the perceived danger for the group of neutral objects was significantly lower than the perceived danger for the group of dangerous objects [*M* = 1.79 vs. *M* = 6.23, *t*(19) = 6.01, *p* < 0.001]. This implies that reaching toward and grasping a neutral object can be considered as a neutral action while reaching toward and grasping a dangerous object can be considered a dangerous action.

**Figure 1:**
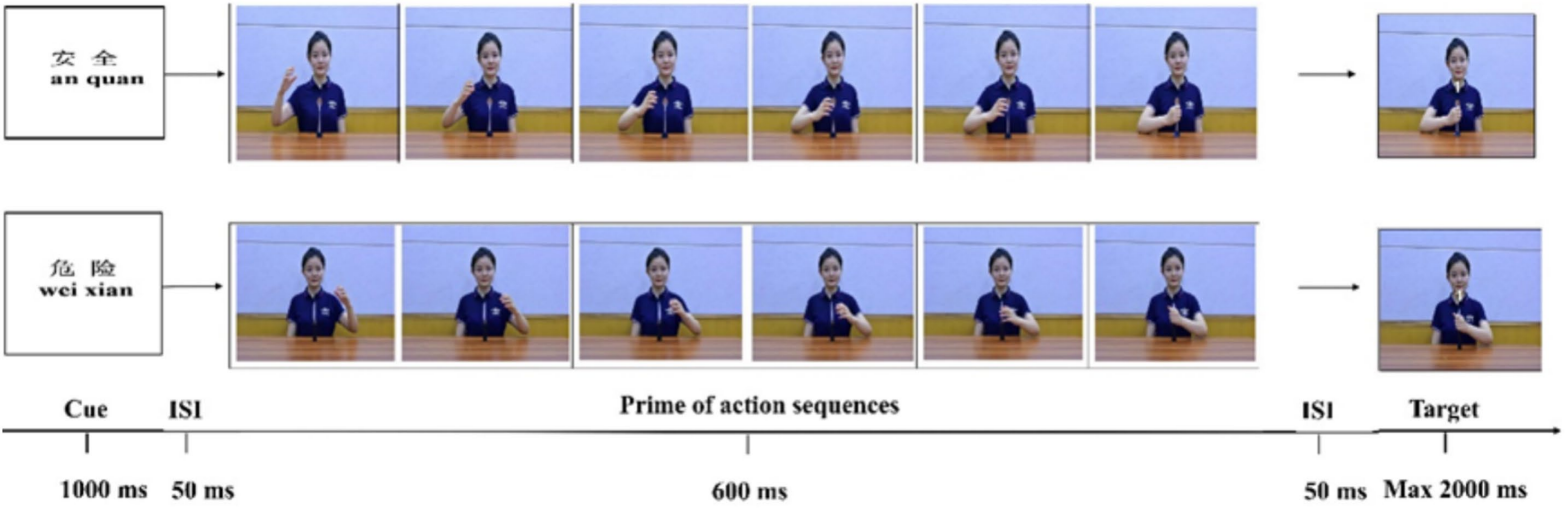
Examples of stimuli used in Experiment 1. Taking reactions (responding to ‘1’ by index finger of the left hand and ‘2’ by index finger of the right hand) as a reference, the above group of pictures depicts a condition of neutral action, with present cue and congruent condition, whereas the below group of pictures depicts a condition of dangerous action, with present cue and incongruent condition.

The word cues were the Chinese characters for ‘danger’ and ‘safe’ (present condition), which subtended 0.9 × 0.9° of visual angle. The ‘danger’ cue corresponded to the dangerous action scenario, while the ‘safe’ cue corresponded to the neutral action scenario. The control condition was a blank screen (absent condition). In order to ensure that the children could understand the meaning of the cues, the corresponding Chinese pinyin was provided below each Chinese character (see Figure 1).

The stimuli were presented on a 17-inch screen, which was placed about 50 cm away from the participants. E-prime1.1 was used to control the presentation of the stimuli and collect the experimental data. Responses were recorded using a keyboard. Experiments 1a and 1b used the same materials; the only difference between these two experiments was the age of the participants (children vs. adults).

#### Procedure

At the start of each trial, a fixation point was presented at the center of the screen for 500 ms. After this, a 1,000 ms cue (word cue or blank screen) would be displayed on the screen. Fifty ms later, participants would be shown a series of pictures depicting an agent’s hand reaching and grasping an object. This process lasted 600 ms. After a blank screen of 50 ms, a number (‘1’ or ‘2’) appeared on the same picture as the final picture, and this was presented for a maximum of 2000 ms (see Figure 1).

Participants were asked to judge the number appearing on the final picture. If the number was ‘1’, participants should press the ‘A’ key on the keyboard with their left index finger; if it was ‘2’, they should press the ‘L’ key on the keyboard with their right index finger. All participants were encouraged to respond as quickly as possible.

The response mode was matched among the participants: half of the participants judged according to the method described above, and the other half made the keypress judgments oppositely. If the number was ‘1’, participants should press the ‘L’ key on the keyboard with their right index finger; if it was ‘2’, they should press the ‘A’ key on the keyboard with their left index finger.

#### Design

Both Experiment 1a and Experiment 1b included four blocks, where each block contained 64 trials, giving a total of 256 trials. In order to eliminate the gender factor of agents (Zhao et al., 2023), the same female agent demonstrated the same action in two blocks of trials, and the same male agent demonstrated the same action in the other two blocks of trials. Experiment 1a adopted a 2 (word cue: present or absent) × 2 (action context: neutral or dangerous) × 2 (congruency between the participant’s response hand and the agent’s hand: congruent or incongruent) factor design. The word cue was the between-participants factor, and the others were the within-participants factors. As a result, half of the children were treated with word cues, with 128 trials each for dangerous and safe cues; the other half received the cue-free treatment, with 256 trails.

A neural context refers to the agent operating a neutral object, while the dangerous context refers to the agent operating a dangerous object. The results of previous studies indicated that the imitation between two hands is performed in a mirrored manner (Bekkering et al., 2000; Koski et al., 2003; Liepelt et al., 2010). Thus, in Experiment 1, the congruent condition referred to the specular manner between the agent’s action hand and the participant’s response hand. The incongruent condition referred to the anatomical manner between the agent’s action hand and the participant’s response hand.

Before the experiment formally began, 20 practice trials were presented to familiarize the participants with the experimental task and response method. Participants could take a break after each block.

### Results

The accuracy rates of Experiment 1a and 1b were 97.6 and 98.7%, respectively. There was no correlation between the error rates and response times [Experiment 1a: Pearson’s *r* (38) = −0.51, *p* = 0.759; Experiment 1b: Pearson’s *r* (38) = 0.22, *p* = 0.186], which eliminates the possibility of a trade-off between accuracy and speed. Only the correct trials were counted in the subsequent analysis, and the trials with response times greater than 1,000 ms or less than 200 ms were excluded (Experiment 1a: 6.4%; Experiment 1b: 4.8%). The remaining data were subjected to a 2 (word cue: present or absent) × 2 (action context: neutral or dangerous) × 2 (congruency between the participant’s response hand and the agent’s hand: congruent or incongruent) repeated measures ANOVA.

### Experiment 1a

**Figure 2:**
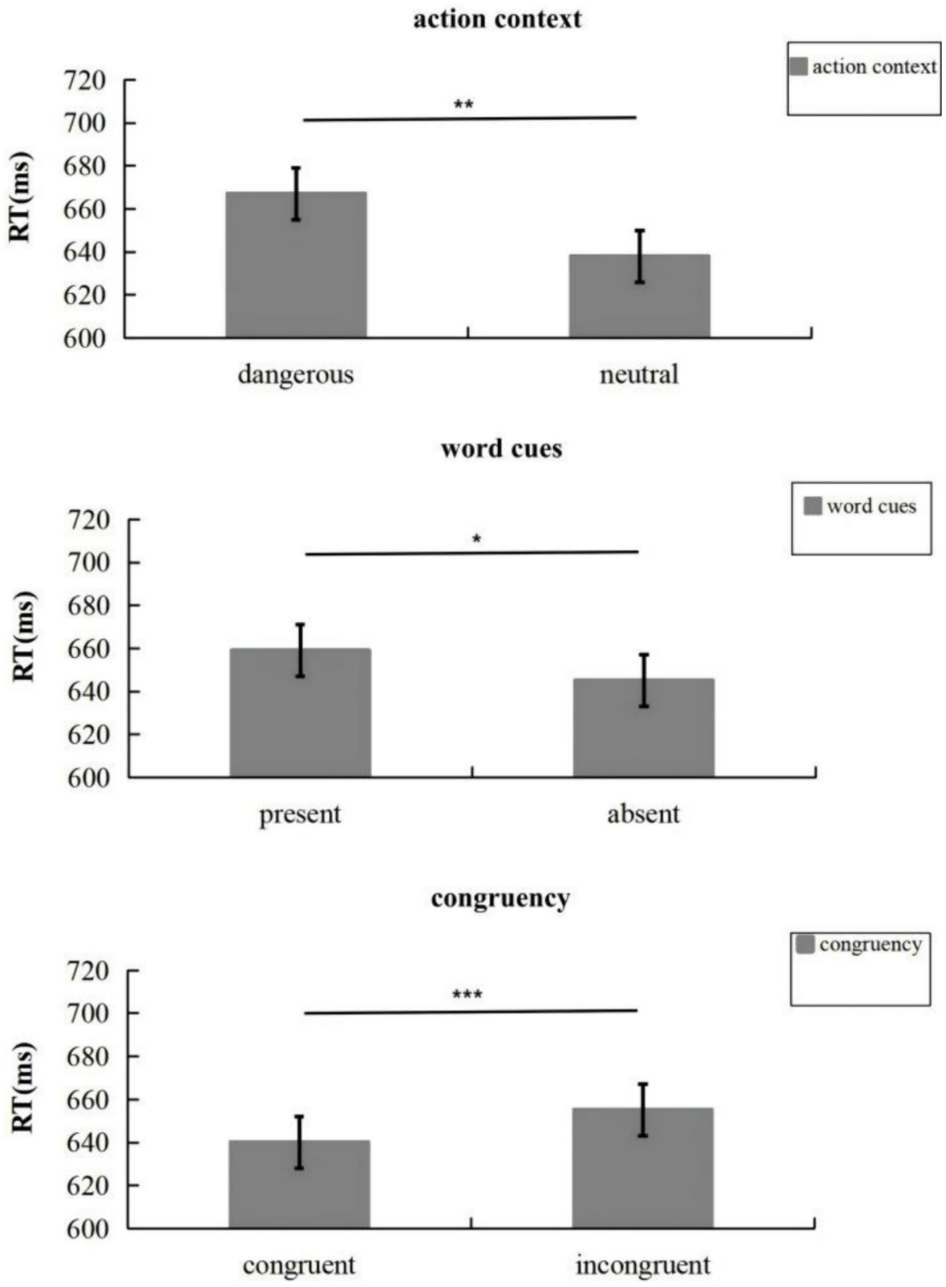
Mean response time (with standard errors) for children in Experiment 1a. Error bars denote standard errors. *p < 0.05; **p < 0.01; ***p < 0.001.

As shown in Figure 2, the ANOVA found that the main effect of the action context was significant, *F*(1, 37) = 10.47, *p* = 0.003, *η^2^* = 0.22, such that individuals’ response time in the dangerous action scenario (667 ms) was slower than their response time in the neutral action scenario (638 ms). Word cues exhibited a significant main effect, *F*(1, 37) = 5.53, *p* = 0.024, *η^2^* = 0.14: response time was quicker without word cues (present: 659 ms, absent: 645 ms). The main effect of congruency between response hands and the agent’s hand was also significant, *F*(1, 37) = 91.36, *p* < 0.001, *η^2^* = 0.71: when the response hands were congruent with the agent’s hand, the response time was shorter (640 ms) than in the incongruent condition (665 ms). The results are summarized in Figure 2; no interaction effects were found, *ps* > 0.10.

### Experiment 1b

The ANOVA found that the main effect of the action context was significant, *F* (1, 37) = 9.50, *p* = 0.004, *η^2^* = 0.20, such that individuals’ response time in the dangerous action scenario (446 ms) was slower than their response time in the neutral action scenario (434 ms). Word cues had a significant main effect, *F* (1, 37) = 8.21, *p* = 0.007, *η^2^* = 0.19: response time was quicker with word cues (present: 435 ms, absent: 445 ms). The main effect of congruency between response hands and the agent’s hand was also significant, *F*(1, 37) = 35.09, *p* < 0.001, *η^2^* = 0.49: when the response hands were congruent with the agent’s hand, the response time was shorter (434 ms) than in the incongruent condition (446 ms). The interaction between the action context and congruency between response hands and the agent’s hand was significant, *F*(1, 37) = 9.28, *p* = 0.004, *η^2^* = 0.20; the interaction between word cues and congruency between response hands and the agent’s hand was also significant, *F*(1, 37) = 5.50, *p* = 0.024, *η^2^* = 0.13; however, the interaction between word cues and action context was not significant. The three-way interaction was similarly significant, *F*(1, 37) = 7.89, *p* = 0.009, *η^2^* = 0.18.

**Figure 3:**
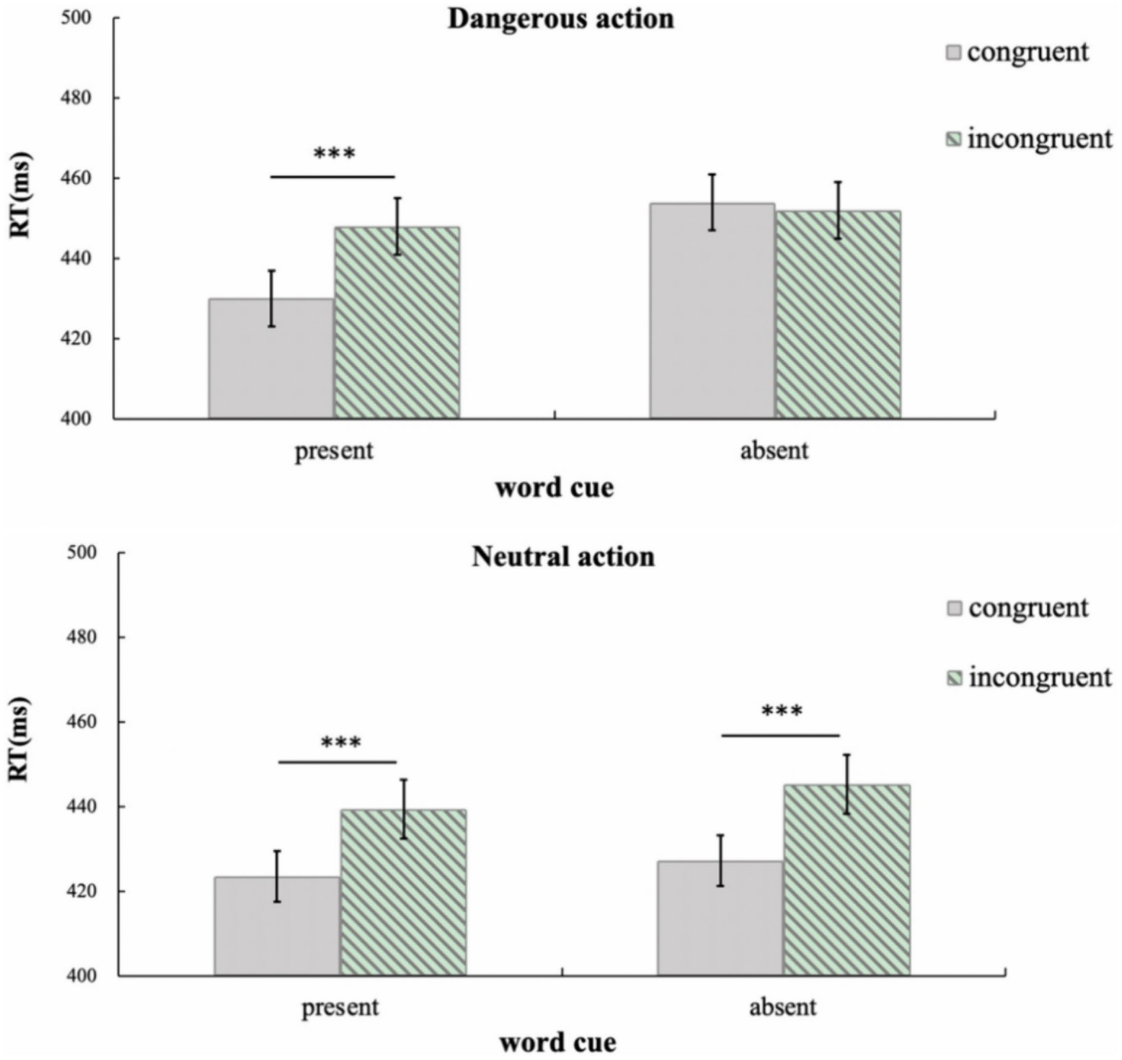
Mean response time (with standard errors) for adults in Experiment 1b. Error bars denote standard errors. *p < 0.05; **p < 0.01; ***p < 0.001.

In order to better compare imitation effect in the dangerous action context and neutral action context, the data were reanalyzed using a 2 × 2 ANOVA (word cue: present, absent; congruency between response hands and the agent’s hand: congruent, incongruent), as shown in Figure 3.

In dangerous action contexts, the main effect of word cues was significant, *F*(1, 37) = 8.59, *p* = 0.008, *η^2^* = 0.19, and the main effect of congruency between response hands and the agent’s hand was also significant, *F*(1, 37) = 11.60, *p* = 0.002, *η^2^* = 0.24. The interaction between the two factors was significant, *F*(1, 37) = 13.74, *p* = 0.001, *η^2^* = 0.27. *Post hoc* paired *t*-tests indicated that in the presence of word cues, imitation effect was detected, i.e., response time was shorter when the response hands were congruent with the agent’s hand (430 ms) than in the incongruent condition (446 ms), *t*(37) = 4.80, *p* < 0.001; in the absence of word cues, there was no imitation effect, i.e., there was no difference in response time between the congruent (454 ms) and incongruent (452 ms) conditions, *t*(37) = 0.66, *p* = 0.515.

In neutral action contexts, the main effect of word cues was not significant, *F*(1, 37) = 0.97, *p* = 0.332, *η^2^* = 0.03. However, the main effect of congruency between response hands and the agent’s hand was significant, *F*(1, 37) = 34.82, *p* < 0.001, *η^2^* = 0.48: when the response hands were congruent with the agent’s hand, the response time (425 ms) was shorter than in the incongruent condition (442 ms). There was no significant interaction between the two factors, *F*(1, 37) = 0.16, *p* = 0.696, *η^2^* = 0.004.

### Brief discussion of experiment 1

Experiment 1 demonstrated that children always showed imitation effect; that is to say, for children, neither action condition nor word cue has an effect on the inhibition of imitation effect. While the only difference between Experiment 1b and Experiment 1a was that there was no imitation effect of dangerous action condition without a dangerous word cue. Therefore, for adults, observing dangerous actions can directly restrain imitation effect.

The results did not support the findings of Zhao (2020) on affordance that word cues can help children appropriately manipulate objects. Due to their strong curiosity and inadequate control ability (Zhao, 2020), and the tendency to over-imitate (Stengelin et al., 2023; Whiten et al., 2009), children show the imitation effect of dangerous actions in both the presence and absence of word cues. In addition, an interesting result of this experiment was that adults tend to imitate dangerous actions in the presence of word cues. An explanation of this result is provided in the general discussion section.

## Experiment 2

### Method

#### Participants

The other 76 participants were recruited for this experiment (none of the above subjects participated in Experiment 1). Thirty eight elementary school students (18 males), aged between 7 and 13 years old, participated in Experiment 2a; 38 college students (18 males), aged between 21 and 25 years old, participated in Experiment 2b. All of the participants were recruited at random. All participants were right-handed, and they had normal or corrected-to-normal eyesight and had not participated in similar experiments.

#### Materials

**Figure 4:**
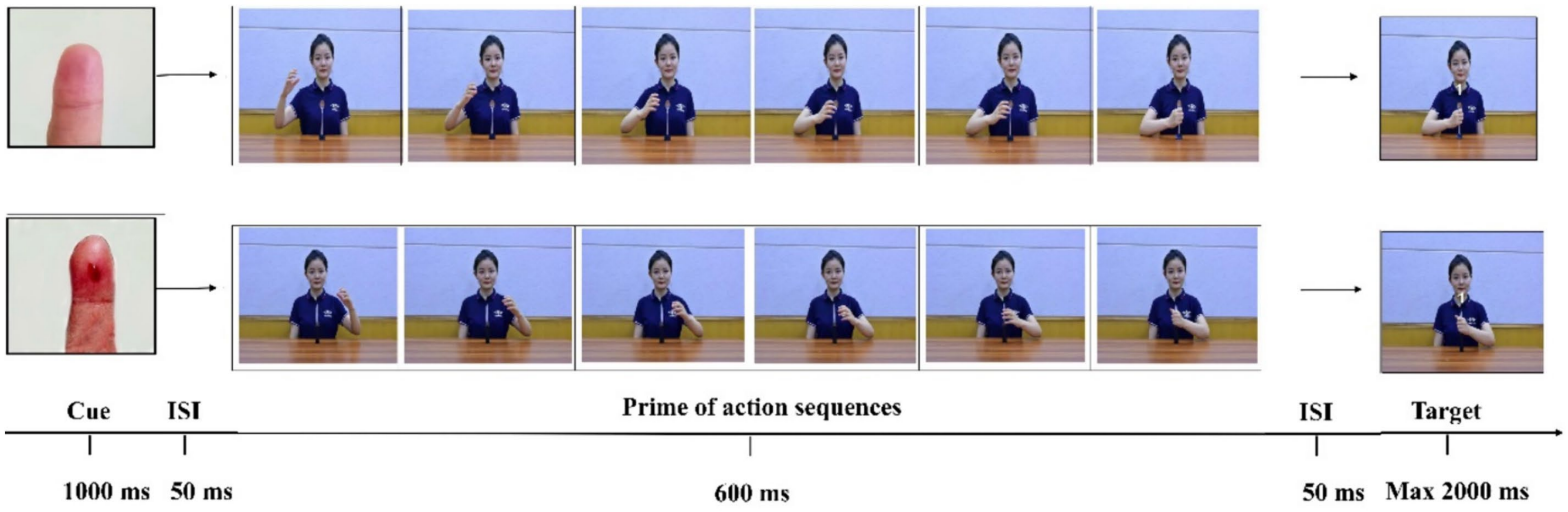
Examples of stimuli used in Experiment 2. Taking reactions (responding to “1” index finger of the left hand and ‘2’ by index finger of the right hand) as a reference, the above group of pictures depicts a condition of neutral action, with present cue and congruent condition, whereas the below group of pictures depicts a condition of dangerous action, with present cue and incongruent condition.

The experimental materials and equipment were essentially the same as those in Experiment 1, except that the word cues were changed to picture cues (see Figure 4). The cue in the dangerous action contexts was an image of bloody fingers, while the cue in the neutral action contexts was an image of usual fingers. The control condition was a blank screen (absent condition).

#### Procedure

The procedure of Experiment 2 was the same as Experiment 1.

#### Design

The design was the same as that of Experiment 1.

### Results

The accuracy rates of experiment 2a and experiment 2b were 97.5 and 98.6%, respectively. Similar to the results of experiment 1, there was no correlation between error rates and response times [Experiment 2a: Pearson’s *r* (38) = 0.56, *p* = 0.737; Experiment 2b: Pearson’s *r* (38) = −0.17, *p* = 0.310], which eliminates the possibility of a speed-accuracy trade-off. Only correct trials were counted in the subsequent analysis, and trials with a response time greater than 1,000 ms or less than 200 ms were excluded. The remaining data were subjected to a 2 (picture cue: present or absent) × 2 (action context: neutral or dangerous) × 2 (congruency between the participant’s response hand and the agent’s hand: congruent or incongruent) repeated measures ANOVA.

### Experiment 2a

The ANOVA found that the main effect of the action context was significant, *F*(1, 37) = 26.77, *p* < 0.001, *η^2^* = 0.42: individuals reacted more slowly to the dangerous action context (677 ms) than to the neutral action context (649 ms). There was a significant main effect of picture cue, *F*(1, 37) = 28.62, *p* < 0.001, *η^2^* = 0.44, such that response time was longer with picture cues (680 ms) than without picture cues (646 ms). The main effect of congruency between response hands and the agent’s hand was also significant, *F*(1, 37) = 72.46, *p* < 0.001, *η^2^* = 0.66: when the response hands were congruent with the agent’s hand, the response time (652 ms) was shorter than in the incongruent condition (674 ms). There was a significant interaction between the presence or absence of picture cues and the congruency between response hands and the agent’s hand, *F*(1, 37) = 6.94, *p* = 0.012, *η^2^* = 0.16. The interaction of the three factors was significant, *F*(1, 37) = 5.35, *p* = 0.026, *η^2^* = 0.14, and there were no other main or interaction effects, *ps* > 0.1.

**Figure 5:**
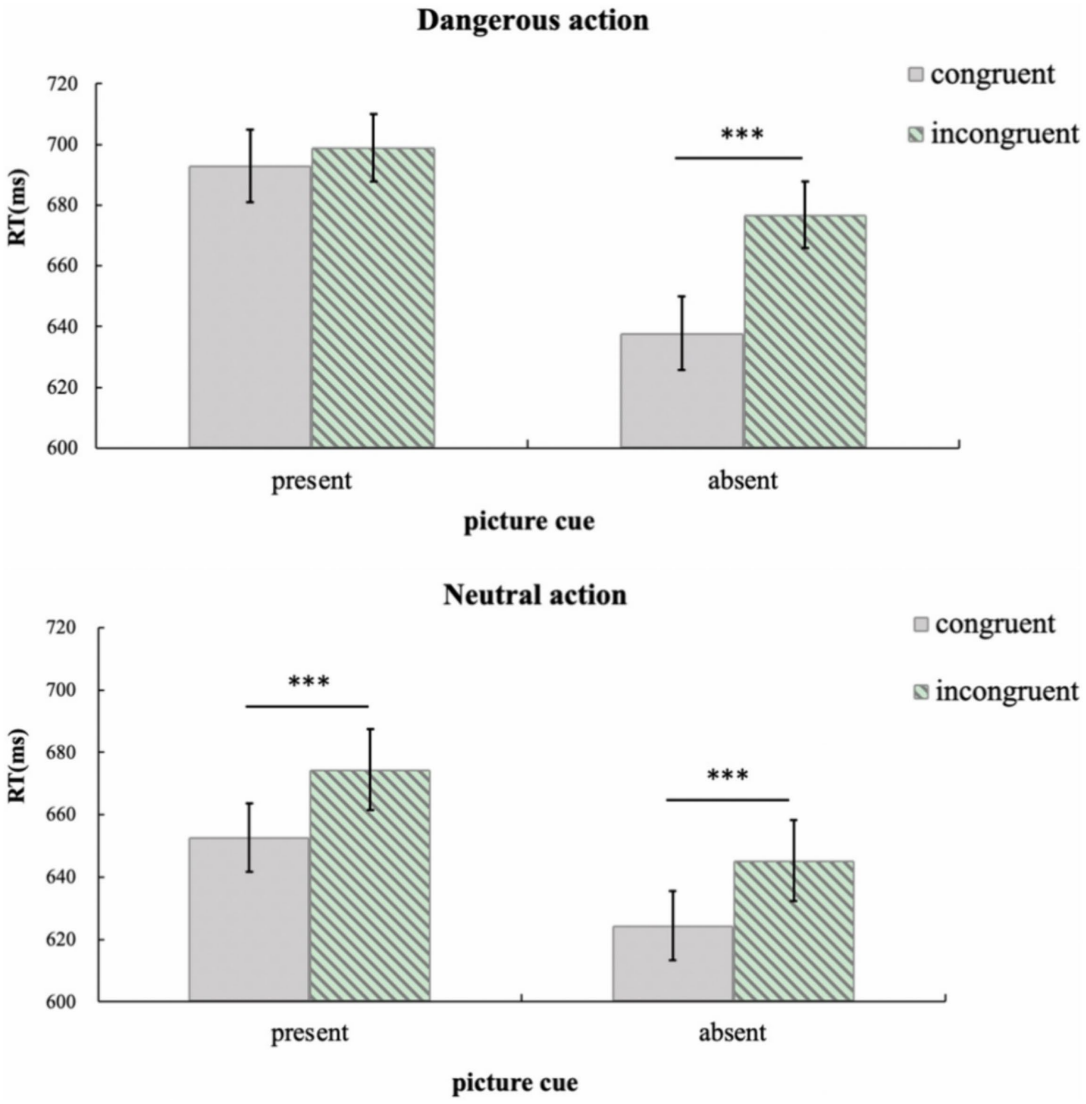
Mean response time (with standard errors) for children in Experiment 2a. Error bars denote standard errors. *p < 0.05; **p < 0.01; ***p < 0.001.

In order to better compare imitation effect in the dangerous action and the neutral action contexts, the data were re-analyzed using a 2 (picture cue: present, absent) × 2 (congruency between response hand and agent’s hand: congruent, incongruent) repeated measures ANOVA. Results are shown in Figure 5.

In dangerous action contexts, the main effect of the picture cues was significant, *F*(1, 37) = 15.63, *p* < 0.001, *η^2^* = 0.30, as was the main effect of congruency between the response hands and the agent’s hand, *F*(1, 37) = 28.50, *p* < 0.001, *η^2^* = 0.44. The interaction between the two was also significant, *F*(1, 37) = 11.35, *p* = 0.002, *η^2^* = 0.24. *Post hoc* paired *t*-tests showed that when there was no picture cue, imitation effect was observed, i.e., in the congruent condition, the response time was shorter (638 ms) than in the incongruent condition (677 ms), *t*(37) = 4.76, *p* < 0.001; when picture cues were presented, there was no imitation effect, i.e., there was no difference in the response time in the congruent (693 ms) and incongruent (699 ms) conditions, *t*(37) = 1.46, *p* = 0.152.

In neutral action contexts, the main effect of picture cues was significant, *F*(1, 37) = 11.79, *p* = 0.001, *η^2^* = 0.24, such that response time was longer with picture cues (664 ms) than without (639 ms). The main effect of congruency between response hands and the agent’s hand was also significant, *F*(1, 37) = 26.76, *p* < 0.001, *η^2^* = 0.42: when the response hands were congruent with the agent’s hand, the response time (639 ms) was shorter than in the incongruent condition (660 ms). The interaction between the two factors was not significant, *F*(1, 37) = 0.01, *p* = 0.917, *η^2^* = 0.00.

### Experiment 2b

The ANOVA found that the main effect of the action context was significant, *F*(1, 37) = 6.21, *p* = 0.017, *η^2^* = 0.15: individuals reacted more slowly to the dangerous action context (451 ms) than to the neutral action context (438 ms). The main effect of congruency between response hands and the agent’s hand was also significant, *F*(1, 37) = 34.14, *p* < 0.001, *η^2^* = 0.48: when the response hands were congruent with the agent’s hand, the response time (437 ms) was shorter than in the incongruent condition (452 ms). There was a significant interaction between the picture cues and the congruency between response hands and the agent’s hand, *F*(1, 37) = 6.91, *p* = 0.012, *η^2^* = 0.16. The interaction of the three factors was significant, *F*(1, 37) = 6.50, *p* = 0.015, *η^2^* = 0.15.

**Figure 6:**
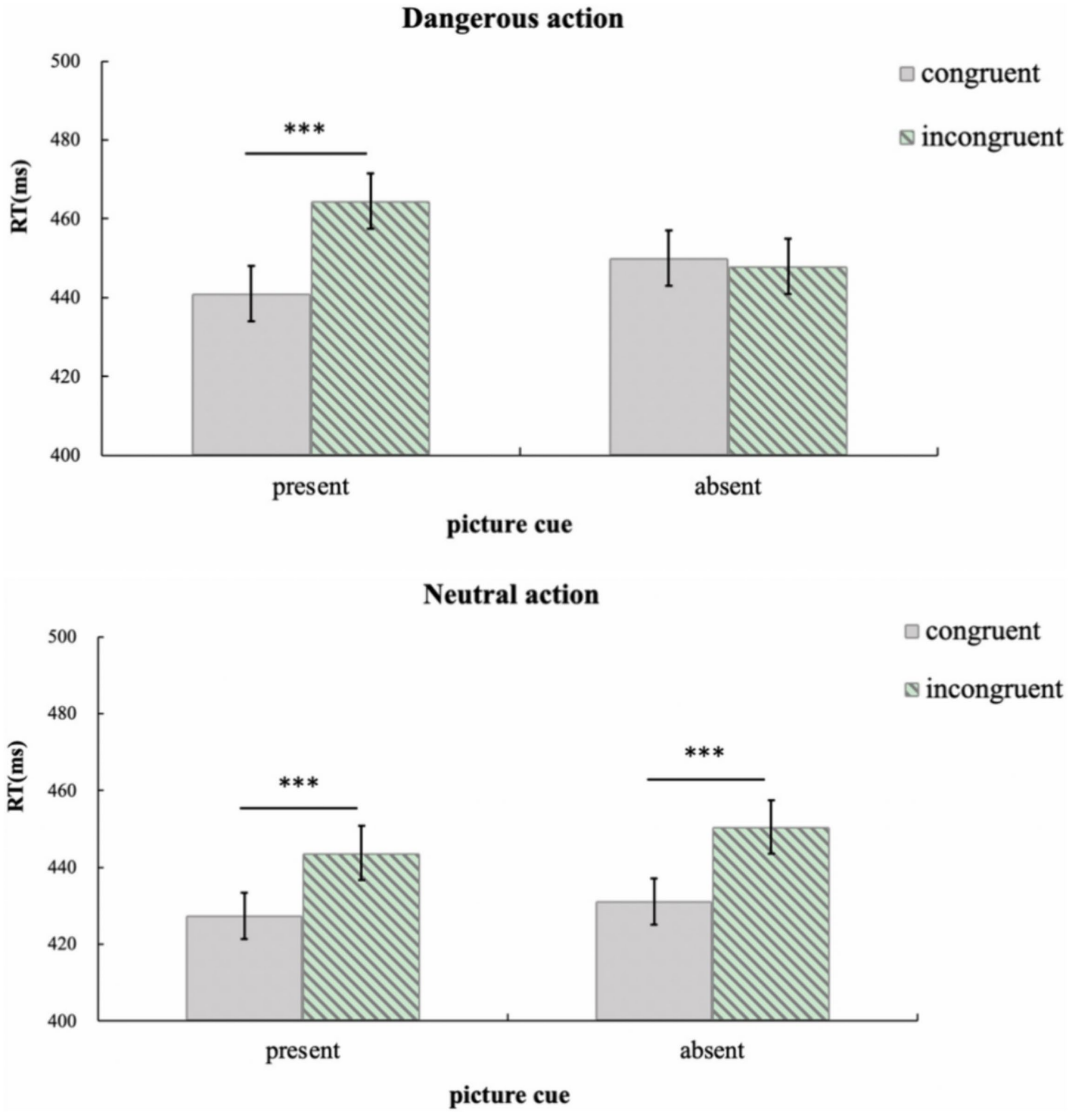
Mean response time (with standard errors) for adults in Experiment 2b. Error bars denote standard errors. *p < 0.05; **p < 0.01; ***p < 0.001.

In order to better compare imitation effect in the dangerous action and the neutral action contexts, the data were re-analyzed using a 2 (picture cue: present, absent) × 2 (congruency between response hand and agent’s hand: congruent, incongruent) repeated measures ANOVA. Results are shown in Figure 6.

In dangerous action contexts, the main effect of congruency between response hands and the agent’s hand was significant, *F*(1, 37) = 11.39, *p* = 0.002, *η^2^* = 0.24. There was a significant interaction between the picture cues and the congruency between response hands and the agent’s hand, *F*(1, 37) = 17.12, *p* < 0.001, *η^2^* = 0.32. *Post hoc* paired t-tests indicated that when picture cues were presented, imitation effect was observed, i.e., the response time was shorter when the response hands were congruent with the agent’s hand (441 ms) than in the incongruent condition (465 ms), *t*(37) = 5.18, *p* < 0.001; when there was no picture cue, there was no imitation effect, there was no difference between the response time in the congruent (450 ms) and incongruent (448 ms) conditions, *t*(37) = 0.48, *p* = 0.637.

In neutral action contexts, the main effect of congruence between response hands and the agent’s hand was also significant, *F*(1, 37) = 24.12, *p* < 0.001, *η^2^* = 0.40: when the response hands were congruent with the agent’s hand, the response time (429 ms) was shorter than in the incongruent condition (447 ms). The main effect of picture cues was not significant, *F*(1, 37) = 1.43, *p* = 0.239, *η^2^* = 0.04. The interaction between the two factors was not significant, *F*(1, 37) = 0.15, *p* = 0.705, *η^2^* = 0.004.

### Brief discussion of experiment 2

Compared with Experiment 1a, the difference between Experiment 2a was that there was no imitation effect in the case of dangerous action conditions with cues (picture type). This suggests that, for children, picture cues can help inhibit imitation effect under dangerous action conditions. This result shows that compared with textual stimuli, the information conveyed by pictorial stimuli is more intuitive and easier for children to process. This May be a reasonable explanation for the difference in the results of Experiment 1a and Experiment 2a.

Consistent with the results of Experiment 1b, in Experiment 2b, there was no imitation effect of dangerous action conditions without picture cues. This once again proves that, for adults, observing the dangerous action suppresses imitation effect. In addition, an interesting result also existed in this experiment: adults tend to imitate dangerous actions in the presence of picture cues. An explanation of this result is provided in the general discussion section.

## General discussion

The observed actions of agents in a large number of studies on imitation effect have not involved dangerous actions. However, in daily life, we have also observed some dangerous actions, such as highly difficult acrobatic performances. In China, when the audience watching these acrobatics of TV programs, they will find that in a corner of the screen that read “professional action, please do not imitate.” Therefore, the main purpose of our study was to explore whether dangerous cues could help children or adults suppress the tendency to imitate dangerous actions.

Based on the results of the two experiments (see Table 1), the current study manifests that picture (not word) cues can help children suppress the tendency to imitate dangerous actions; for adults, observing dangerous actions can directly inhibit imitation effect; In adults, observing dangerous actions in the absence of cues can directly inhibit the imitation effect; while, when presented with a picture or word cue, adults always showed an imitation effect on dangerous actions. Finally, under the condition of agents demonstrate neutral action, adults and children always showed the imitation effect.

**Table 1:** Summary of results on inhibition of imitation effect.

	Dangerous action condition			Neutral action condition		
	Word cue	Picture cue	Absent	Word cue	Picture cue	Absent
Children	○	√	○	○	○	○
Adults	○	○	√	○	○	○

√ Represents successfully inhibit imitation effect; ○ stands for imitation effect, which means inhibition fails.

When presented with a danger cue, only the children performed in line with the affordance studies (Myachykov et al., 2013; Zhao, 2020): danger cues did help them respond appropriately and inhibit imitation effect. Ulteriorly, picture instead of word prompts help children to construct the meaning of dangerous information and prevent the tendency to imitate dangerous actions in a top-bottom manner (Kanske and Kotz, 2007). This means that for children, picture cues May have an advantage over word cues in suppressing the tendency to imitate dangerous behaviors.

In the absence of cues, adults suppressed the tendency to imitate dangerous behavior, which was consistent with the results of Zhao et al. (2023). Surprisingly, adults fail to suppress the tendency to imitate dangerous behaviors when presented with dangerous cues (both picture and word). This May be because adults’ dangerous awareness is activated when dangerous cues are presented, and they May pay attention to the source of the danger information, such as the blade of a knife (Zhao, 2020). This makes them unconsciously imitate the behavior of the agent, leading to suppression failure.

In the context of observing neutral actions, both children and adults consistently exhibited imitation effects, irrespective of the types of cues provided to them. This consistent display of imitation effects, regardless of cue type, further substantiates the empirical support for the MNS (mirror neuron system) hypothesis, as proposed by Rizzolatti and Sinigaglia (2016). This evidence underscores the robustness of the MNS in facilitating action imitation across different age groups and cue conditions.

An alternative hypothesis should be considered in this study: the results might stem more from an inhibition of the affordance (stimulus–response) facilitation than from the imitation mechanism. As Brass and colleagues have indicated, imitation can be measured using the stimulus–response compatibility (SRC) paradigm. However, the Brass paradigm did not involve any objects. In their study, participants were asked to observe task-irrelevant finger movements while task-relevant stimuli appeared next to the fingers, and to use the corresponding fingers to provide task-related responses (Brass et al., 2000). Brass argued that imitation might be distinct from affordance facilitation, particularly when the action to be imitated is object-centered, especially when the object is recognized, and the action goal is easily identified and known from experience. Conversely, when no object is involved (but the actions are still meaningful to the observer), Brass suggested that compatibility triggers imitation. When the action does not involve an object and is meaningless to the observer, the process of imitation requires mental Decomposition and recomposition of the observed action (Tessari and Rumiati, 2002). We acknowledge that this idea can partially explain imitation. Bertenthal et al. (2006) found that spatial compatibility contributes to the imitation effect to some extent. Nonetheless, this experiment cannot entirely rule out this hypothesis. Future research is needed to explore the underlying mechanisms in real operational scenarios.

This study found that presenting danger cues with pictorial warnings can help children better inhibit their tendency to imitate dangerous actions. This suggests that pictorial warnings, by providing salient visual cues, can enhance children’s cognitive control abilities, particularly in regulating and controlling behavioral responses through external cues. Additionally, the findings of this study provide empirical evidence for improving safety education and accident prevention. By using pictorial warning signs in everyday life, such as in homes, schools, and public places, it is possible to more effectively remind children to stay away from potential dangers, thereby reducing the occurrence of accidental injuries. By focusing on the superior impact of pictorial cues, educators and parents can develop more effective strategies for communicating safety information to children, thereby fostering a safer environment for their growth and development. In future studies, we can continue to explore the role of video warnings in inhibiting children’s tendency to engage in dangerous actions.

## Data Availability

The original contributions presented in the study are included in the article/supplementary material, further inquiries can be directed to the corresponding author.
